# Ethics in times of physical distancing: virtual training of ethical competences

**DOI:** 10.3205/zma001424

**Published:** 2021-01-28

**Authors:** Susanne Michl, Johannes Katsarov, Henriette Krug, Annette Rogge, Tobias Eichinger

**Affiliations:** 1 Charité – Universitätsmedizin Berlin, Institut für Geschichte der Medizin und Ethik in der Medizin, Berlin, Germany; 2 Universität Zürich, Ethik-Zentrum, Zurich, Switzerland; 3 Medical School Hamburg, Fakultät für Gesundheitswissenschaften, Hamburg, Germany; 4 Universitätsklinikum Schleswig-Holstein, Campus Kiel, Medizinethik, Institut für Experimentelle Medizin, Kiel, Germany; 5 Universität Zürich, Institut für Biomedizinische Ethik und Medizingeschichte, Zurich, Switzerland

**Keywords:** medical ethics, ethics didactics, blended learning, interactivity

## Abstract

Ethics teaching in medicine, nursing and other health care professions does not only consist of knowledge transfer that can be easily implemented digitally. Rather, it focuses on specific ethical competences (such as arguing and articulating one's own moral position) and attitudes (such as empathic patient orientation, critical self-reflection, and ambiguity tolerance), for whose development interactive formats are superior. Competence-oriented ethical learning goals are important for the development of professionalism, but require time, space and personal exchange. Due to contact restrictions and the widespread cancellation of (face-to-face) courses in the wake of the corona pandemic, ethics teaching was forced to keep its distance in many places, which posed great challenges. This article is based on an exchange of experiences from members of the working group *ethik learning* of the Academy for Ethics in Medicine about ethics teaching in times of physical distancing. Recommendations will be given on how ethical competence can be successfully taught in the context of exclusively digital teaching. Starting with the question what is at risk of being lost in digital teaching, the potentials of digital formats are explored and illustrated with concrete practical examples. Beyond ethics teaching, the article also aims to provide ideas and suggestions for other specialist and cross-sectional areas where interactive formats are central.

## Zusammenfassung

Ethik-Lehre in Medizin, Pflege und anderen Gesundheitsberufen besteht nicht allein aus Wissensvermittlung, die digital gut umzusetzen ist. Im Mittelpunkt stehen vielmehr spezifische ethische Fertigkeiten (etwa Argumentieren und Artikulieren der eigenen moralischen Position) und Haltungen (etwa empathische Patientenorientierung, kritische Selbstreflexion und Ambiguitätstoleranz), für deren Entwicklung interaktive Formate besser geeignet sind. Kompetenzorientierte ethische Lernziele sind für die Entwicklung des professionellen Rollen- und Selbstverständnisses wichtig, erfordern jedoch Zeit, Raum und vor allem den persönlichen Austausch. Durch die Kontaktbeschränkungen und den flächendeckenden Ausfall von (Präsenz-)Lehrveranstaltungen im Zuge der Corona-Pandemie war die Ethik-Lehre vielerorts gezwungen, auf Abstand zu gehen, was große Herausforderungen mit sich brachte. Der Beitrag basiert auf einem Erfahrungsaustausch von Mitgliedern der Arbeitsgruppe *ethik learning* der Fachgesellschaft *Akademie für Ethik in der Medizin* über Ethik-Lehre in Zeiten von *physical distancing*. Es werden Empfehlungen gegeben, wie ethische Kompetenzvermittlung im Rahmen ausschließlich digital stattfindender Lehre gelingen kann. Ausgehend davon, was bei digitalem Unterricht verloren zu gehen droht, werden die Potenziale digitaler Formate ausgelotet und mit konkreten Praxisbeispielen illustriert. Über die Ethik-Lehre hinaus möchte der Beitrag auch Ideen und Anregungen für andere Fach- und Querschnittsbereiche geben, bei denen interaktive Formate zentral sind.

## Introduction

Teaching ethics requires more than instructing people on normative theory. Judgement, argumentation, ambiguity tolerance, and a reflection of one‘s own values and emotions shape professionals’ identity, but need time, continuity, and space to develop. Meta-analyses show that interactive discourse is indispensable for this [1]. Although digital teaching formats are particularly well suited for conveying theoretical knowledge [2], they often fail to convey action-related competences and professional attitudes that are largely based on the reflection of experiences, actions, and feedback [3], [4]. The large number of learning and competence goals in ethics teaching suggests a blended-learning approach in which interactive face-to-face teaching is combined with digital opportunities for self-study in a meaningful way. 

## Methodology

The article presents tips and tools for the training of ethical competencies in digital teaching settings, which were collected and discussed via video conference by 13 members of the* ethik learning* working group of the Academy for Ethics in Medicine in July 2020. 

## Results: tips and tools

If teaching takes place exclusively in digital form, this reduces the spontaneity and agility of group-dynamic processes, as well as the commitment of the students to represent their personal values. Individual and context-specific forms of articulation and feedback threaten to be lost. The socially experienced emotionality and vividness of a learning community are thus reduced overall. In addition, it seems more difficult to directly control learning processes in digital teaching formats. 

But how can interactive teaching and the training of ethical competence be successful, even if there is no more face-to-face teaching?

To make the best possible use of digital tools, different formats should be combined in such a way that both synchronous and asynchronous communication and interaction in the learning group are enabled. Since an exclusively digital setting inevitably poses a greater focus on self-study and group dynamic processes are more restricted, it is all the more important to activate individual learners in a targeted and varied manner in all phases of the learning process. However, it is hardly possible to activate the learners by simply switching to read-in lectures with frontally presented content. In order to design learning processes in the digital world accordingly, tried and tested modules are now available that enable experience-based ethics learning:

### Game based learning

The Serious Moral Game “uMed: Your Choice” [5]: In this medical ethics video game, a digital role-playing game, students are confronted with difficult clinical-ethical cases from the perspective of an intern. The game consists of various dialogue options that are available in communication situations with patients, superiors, colleagues, nursing staff or relatives, which determine the course of action, whereby one’s choices have consequences that are sometimes difficult to foresee. An essential element of the game’s usage in teaching consists in embedding and reflecting the game experience and the ethical issues it presents in synchronous group units under the guidance of teachers.

#### Simulation-based learning

To practice perspective taking, many ethics classes make use of interactive role play, which can only be transferred to the digital format with restrictions. The tool “Entscheiden und Handeln am Krankenbett” (Deciding and acting at the patient’s bedside) [6] offers an online platform where students are confronted with a clinical-ethical dilemma situation using filmed video sequences where they have to make, communicate and implement a difficult decision. 

#### Learning by writing

In digital exchange, written forms of expression, which are often neglected in medical studies, will once again take on greater importance. In writing, considering, sharing, integrating, and commenting on short and longer texts, the abilities to abstract, articulate and argue will be practiced that are indispensable for moral discourse. In contrast to direct oral exchange, written forms of expression offer time for reflection; they can be shared and commented on, circulated, and, over the course of the semester, form a link between self-study and group-based online phases. Here, new technical possibilities also offer procedures of collaborative writing, which allows a content-focused form of interaction.

#### Discursive learning in the forum

A forum on specific topics encourages students to reflect and discuss, whereby communication can be synchronous or asynchronous. A lively forum exchange can lead to dynamic group processes and a community experience. For the teachers, forum contributions offer insights into the students' learning progress as well as their main areas of interest and conflict, which can be discussed in greater depth in joint online meetings.

#### Collaborative digital learning

To enable spontaneity and agility even in synchronous online lectures, chat functions and survey tools are available for querying initial moral intuitions. Breakout rooms enable work in small groups, for example the analysis of an ethical case with subsequent commentary by another small group in a rotating system or in the plenary. This way, a space can be provided where community is experienced and where students are encouraged to present and justify their own value concepts.

## Discussion and conclusion: accompanying learning processes

Can ethics teaching succeed at a distance? The training of ethical competences thrives on direct exchange, on the clash of different moral positions and, last but not least, on irritation as a starting point for moral reflection. A purely digital teaching of ethics cannot completely replace the learning effects of face-to-face formats. Spontaneity, moments of surprise, and socially experienced emotionality are, as previous experience has shown, partially lost, and cannot be transferred to the digital group room without difficulty. Against this background, the task of the ethics lecturers as process facilitators is primarily to initiate and plan the interaction and group dynamics in the connection of asynchronous and synchronous components. Digital possibilities of game- and simulation-based learning, online chats, and forums as well as the classical focus on written articulations of moral positions – whether on paper or screen – are available.

## Competing interests

The authors declare that they have no competing interests. 
